# 

**Published:** 1889-01

**Authors:** 


Now is the Time to Renew Your Subscription.

VOL. X. JANUARY, 1889. No. 1.

THE

### International Dental Journal:

SUCCESSOR TO THE INDEPENDENT PRACTITIONER.

A MONTHLY PERIODICAL

DEVOTED TO

### DENTAL AND ORAL SCIENCE.

W. XAVIER SUDDUTH, M. D., D. D. S.,
EDITOR.

PUBLISHED BY

THE INTERNATIONAL DENTAL PUBLICATION COMPANY,

51 WEST 37th STREET, NEW YORK CITY,

—AND—

1215 FILBERT STREET, PHILADELPHIA.

$2.50 Per Annum in Advance, Single Copies, 25 Cents.

